# A General Overview on the Hyperbaric Oxygen Therapy: Applications, Mechanisms and Translational Opportunities

**DOI:** 10.3390/medicina57090864

**Published:** 2021-08-24

**Authors:** Miguel A. Ortega, Oscar Fraile-Martinez, Cielo García-Montero, Enrique Callejón-Peláez, Miguel A. Sáez, Miguel A. Álvarez-Mon, Natalio García-Honduvilla, Jorge Monserrat, Melchor Álvarez-Mon, Julia Bujan, María Luisa Canals

**Affiliations:** 1 Department of Medicine and Medical Specialities, Faculty of Medicine and Health Sciences, University of Alcalá, 28801 Alcala de Henares, Spain; cielo.gmontero@gmail.com (C.G.-M.); msaega1@oc.mde.es (M.A.S.); maalvarezdemon@icloud.com (M.A.Á.-M.); natalio.garcia@uah.es (N.G.-H.); jorge.monserrat@uah.es (J.M.); mademons@gmail.com (M.Á.-M.); mjulia.bujan@uah.es (J.B.); 2 Ramón y Cajal Institute of Sanitary Research (IRYCIS), 28034 Madrid, Spain; 3 Cancer Registry and Pathology Department, Hospital Universitario Principe de Asturias, 28806 Alcala de Henares, Spain; 4 Underwater and Hyperbaric Medicine Service, Central University Hospital of Defence—UAH Madrid, 28801 Alcala de Henares, Spain; ecalpel@fn.mde.es; 5 Pathological Anatomy Service, Central University Hospital of Defence—UAH Madrid, 28801 Alcala de Henares, Spain; 6 Immune System Diseases—Rheumatology, Oncology Service an Internal Medicine, University Hospital Príncipe de Asturias, (CIBEREHD), 28806 Alcala de Henares, Spain; 7 ISM, IMHA Research Chair, Former of IMHA (International Maritime Health Association), 43001 Tarragona, Spain; mlcanalsp@gmail.com

**Keywords:** hyperbaric oxygen therapy (HBOT), Hyperoxia, wound healing, antimicrobial properties, Coronavirus Disease-19 (COVID-19)

## Abstract

Hyperbaric oxygen therapy (HBOT) consists of using of pure oxygen at increased pressure (in general, 2–3 atmospheres) leading to augmented oxygen levels in the blood (Hyperoxemia) and tissue (Hyperoxia). The increased pressure and oxygen bioavailability might be related to a plethora of applications, particularly in hypoxic regions, also exerting antimicrobial, immunomodulatory and angiogenic properties, among others. In this review, we will discuss in detail the physiological relevance of oxygen and the therapeutical basis of HBOT, collecting current indications and underlying mechanisms. Furthermore, potential areas of research will also be examined, including inflammatory and systemic maladies, COVID-19 and cancer. Finally, the adverse effects and contraindications associated with this therapy and future directions of research will be considered. Overall, we encourage further research in this field to extend the possible uses of this procedure. The inclusion of HBOT in future clinical research could be an additional support in the clinical management of multiple pathologies.

## 1. Introduction

Hyperbaric oxygen therapy (HBOT) is a therapeutical approach based on exposure to pure concentrations of oxygen (O_2_) in an augmented atmospheric pressure. According to the Undersea and Hyperbaric Medical Society (UHMS), this pressure may equal or exceed 1.4 atmospheres (atm) [1]. However, all current UHMS-approved indications require that patients breathe near 100% oxygen while enclosed in a chamber pressurized to a minimum of 2 ATA [2].

The first documented use of hyperbaric medical therapy was in 1662 by Henshaw, a British physician who placed patients in a container with pressurized air. Interestingly, it was conducted before the formulation of the Boyle-Mariotte Law, which described the relationship between the pressure and volume of a gas, and prior to the discovery of O_2_ by John Priestly over 100 years later [3]. Afterwards, the pathway of HBOT in medical care was retarded by the observation of possible O_2_-derived adverse effects at 100% concentrations by Lavoisier and Seguin in 1789. Years later, in 1872 Paul Bert, considered the “father of the hyperbaric physiology”, described the physiological basis of pressurized air in the human body, also defining the neurotoxic effects of O_2_ in the human body, consequently named the Paul Bert effect [4], followed by the description of the pulmonary toxicity of O_2_ by Lorrain Smith [5]. Simultaneously, a growing interest in the use of HBOT in the treatment of different affections was reported, including treatment for divers who suffered decompression sickness during World War II [6]. Since then, a plethora of studies were prompted, with hundreds of facilities based on HBOT being established at the beginning of the 21st century [7].

Currently, there are 14 approved indications for HBOT, including a wide variety of complications like air embolism, severe anemia, certain infectious diseases or idiopathic sensorial hearing loss. In addition, in the last European Consensus Conference on Hyperbaric Medicine highlighted the use of HBOT as a primary treatment method for certain conditions according to their moderate to high degree of evidence (e.g., after carbon monoxide (CO) poisoning), or as a potential adjuvant to consider in other conditions with a moderate amount of scientific evidence (e.g., Diabetic foot) [8]. In this work we will review in detail the basis of O_2_ as a therapeutical agent and the principles of hyperbaric medicine regarding most relevant applications concerning HBOT, and potential implications for different approaches including COVID-19.

## 2. Physiological Role of Oxygen in the Organism

O_2_ is a frequently disregarded nutrient because of its particular access inside the human body, through the lungs instead of the gastrointestinal tract, typical of all other nutrients [9]. O_2_ is key for human cells to perform so-called aerobic respiration, which takes places in the mitochondria. Here, O_2_ acts as an electron acceptor finally leading to ATP synthesis in a process known as oxidative phosphorylation. From an evolutionary perspective, the uptake of O_2_ was the origin of eukaryotic cells, emerging as a result of an endosymbiotic relationship between prokaryotic cells (archaea and eubacteria) which were capable of using this nutrient [10]. This fact represented an adaptative advantage with regard to those cells unable to utilize it, complex organisms were coevolving with O_2_, thus becoming an essential nutrient for our cells [11].

In a simple manner, O_2_ is introduced in our body by two distinguished process: ventilation, in which gases are transported from the environment to the bronchial tree and diffusion, where an equilibrium in the distribution of O_2_ between alveoli space and blood is reached. Given that the partial pressure of O_2_ (PO_2_) here is low, and rich in carbon dioxide (CO_2_), gas exchange occurs [12]. Simultaneously, the difference in the pressure and volume in the chest wall and lungs are essential to permit the oxygen flow, as atmospheric pressure does not vary at all [13]. Once in the bloodstream, O_2_ is mostly bound to haemoglobin (Hb) in the erythrocytes, and to a little extent in a dissolved form, being systemically distributed. Then, oxygen exchange is produced between the microcirculatory vessels—Not only capillaries, but also arterioles and venules-and the rest of the tissues, due to the different partial pressure of O_2_ and the Hb oxygen saturation (SO_2_), which is also dependant on other variables like temperature, PCO_2_ and pH, among others [14]. If, however there is a lack of oxygen in the tissue it may appear a condition designed as hypoxia. This may be due to low O_2_ content in the blood (Hypoxemia), which may be a consequence of either a disruption in the blood flow to the lungs (Perfusion), airflow to the alveoli (Ventilation) or problems in the gas diffusion in the haemato-alveolar barrier. Furthermore, low blood supply (ischaemia) or difficulties in the O_2_ delivery, may also be responsible for tissue hypoxia [15]. Consequently, within cells there are specific sensors named as Hypoxia-inducible factors (HIF) that under hypoxic conditions will bind to the hypoxia response element (HRE), thereby regulating a wide variety of cellular processes [16]. Occasionally, hypoxia might provide favourable implications for health, for instance during early developmental stages [17] or in the case of intermittent exposures [18]. Nonetheless, hypoxia mostly induce a pathological stress for cells that is closely related with the appearance and progress of a broad spectrum of diseases [19]. As a result, oxygen has been proposed as a potential therapeutic agent for patients undergoing different acute or chronic conditions [20,21]. As targeting cellular hypoxia is a promising, but still an emerging approach [22], clinical management of hypoxia is directed to modulate global hypoxemia and oxygen delivery within the tissues [23]. In this context, HBOT arises as an extraordinary support in the handling of hypoxia and other hypoxia-related phenomena by increasing blood and tissue levels of oxygen [24]. Hereunder, we will describe the principles and mechanisms of action of HBOT, regarding its therapeutical basis and specific considerations of this therapy.

## 3. Principles of Hyperbaric Oxygen Therapy. Therapeutical Basis

As above mentioned, HBOT consist of the supply of pure oxygen under augmented pressure. This procedure is conducted in a monoplace or multiplace chamber if there are only one or various patients undergoing this procedure, respectively. In the first case, the chambers are usually compressed with O_2_ whereas in the second, people breath oxygen individually through a face mask, hood, or an endotracheal tube [25]. In the case of critically ill patients, it seems that multiplace chambers allow a better monitoring of the vital functions in comparison to monoplace chambers, although the use of the latter are also safe and well tolerated by patients [26,27]. Depending on the protocol, the estimated duration of session varies from 1.5 to 2 h and may be performed from one to three times daily, being given among 20 to 60 therapeutical doses depending on the condition [28]. Frequently, this method utilizes between 2 to 3 atms of pressure. Nevertheless, it has also been obtained promising results in some studies from <2 atms (1.5 atms) for certain conditions [29,30], although according to all UHMS currently approved indications it is required a chamber pressurized to a minimum of 2 ATA [2]. Despite some protocols accept the use of 6 atms (i.e., treatment of gas embolism), little benefits are usually reported from >3 atms as it may be associated with a plethora of adverse effects [31]. Moreover, it is not possible to breath pure O_2_ at higher pressures than 2.8 atm, and in those cases it is accompanied with other gases like helium, nitrogen or ozone. The alternative, normobaric oxygen therapy (NBOT), utilizes oxygen at 1 atm of pressure. In comparison with HBOT, NBOT is cheaper and easier to apply, and it could be found in almost all hospitals, as it does not require hyperbaric chambers [32]. However, some studies have reported a reduced efficacy of NBOT in comparison with HBOT [33,34], therefore showing the relevance of HBOT for certain conditions. Conversely, the use of NBOT could be critical for patients suffering from some maladies in absence of HBOT facilities.

The therapeutical basis of hyperbaric oxygenation are consequence of three main factors: (1) By breathing 100% O_2_, a positive gradient is created, hence favouring diffusion for hyperoxigenated lungs to hypoxic tissues; (2) due to the high pressure, O_2_ concentration in the blood raises according to Henry’s Law (the amount of dissolved gas within a liquid is directly proportional to its partial pressure) and (3) it decreases the size of gas bubbles in the blood following Boyle-Mariotte Law and Henry’s Law [6]. In other words, the creation of a hyperbaric environment with pure oxygen permits a significant increment of the oxygen supply to blood (Hyperoxemia) and to the tissues (Hyperoxia) even without the contribution from Hb [35]. Thus, HBOT provides multiple effects in the organism, and it could be used to correct tissue hypoxia, chronic hypoxemia and to aid in the clinical management of different pathological processes including wound healing, necrosis, or reperfusion injuries [36].

Contrary to hypoxia, the human body has not developed any specific adaptation to hyperoxia. Interestingly, the exposure to intermittent hyperoxia, share many of the mediators and cellular mechanisms which are induced by hypoxia. This is called the hyperoxic-hypoxic paradox [37]. Importantly, it does not have to be considered a negative property. As occurring with intermittent hypoxia, the submitting of short-term hyperoxia may provide favourable outcomes in the cell. The explanation resides in a crucial concept in biology, the hormesis, which correlates the type of response obtained with the dose received [38]. From a molecular perspective, high PO_2_ in the tissues may have important implications in the cellular signalling, particularly through increasing the production of reactive oxygen species (ROS) and reactive nitrogen species (RNS). These changes induce multiple effects in the organism, including the synthesis of different growth factors, improving neovascularization or showing immunomodulatory properties, among others, therefore exerting its clinical efficacy [39,40]. Moreover, HBOT upregulates HIF, by ROS/RNS and Extracellular Regulated Kinases (ERK1/ERK2) pathway [37,41]. In the same manner, an excessive production of ROS and RNS due to hyperoxia may lead to the appearance of oxidative stress, DNA damage, metabolic disturbances, endothelial dysfunction, acute pulmonary injury and neurotoxicity [42]. As hyperbaric O_2_ may provide both beneficial and adverse effects, it is essential to balance the different factors to clinically recommend or reject HBOT [43]. Due to the physics of HBOT, it is not easy to design adequate studies and clinical trials to fully endorse its use. Despite this, there are some predictive models that may be an additional tool to evaluate what patients may benefit the most from receiving this therapy, considering distinct therapeutical approaches if necessary [44].

**Figure 1:**
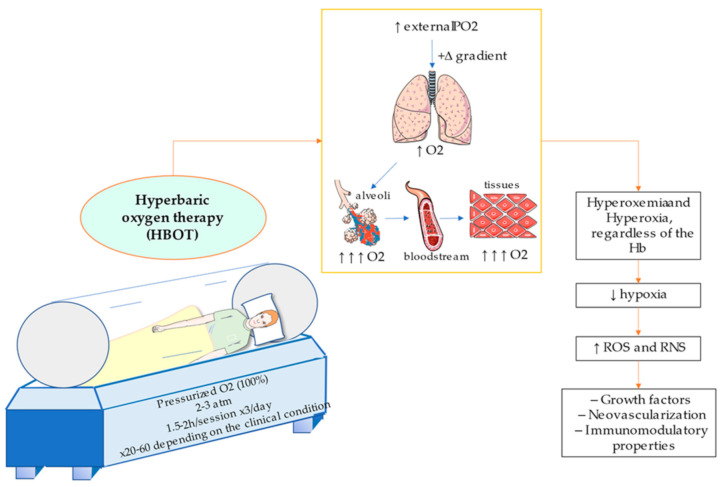
Illustration of a monoplace hyperbaric chamber and the effect of hyperbaric O_2_. Pressurized O_2_ (2–3 atm) at 100% concentration is administered normally during 1.5–2 h per session and repeated three times a day. Depending on the clinical condition sessions vary in number, from 20 to 60. The inhalated air comes from an external elevated PO_2_, hence positive gradient allows higher O_2_ entry, which per diffusion will be higher also in alveoli, bloodstream and therefore there will be greater arrival to tissues. This effect of “hyperoxemia” and “hyperoxia” is independent from haemoglobin (Hb), then will lessen hypoxia in tissues. This will result in a major supply of reactive oxygen species (ROS) and reactive nitrite species (RNS), with a consequent higher expression of growth factors and promotion of neovascularization and enhanced immunomodulatory properties.

In Figure 1 conditions and characteristics of hyperbaric chambers are illustrated, besides the main effect of pressurized O_2_ administration. Below, main applications and translational applications of HBOT will be subsequently discussed, in order to review the actual importance of this procedure in current clinical practice and potential uses.

## 4. Approved Indications for HBOT

Due to the multiple characteristics of HBOT, the possible applications of this procedure are numerous. For instance, HBOT may be used as an urgent treatment for acute pathologies but also as an additional support for chronic diseases [41]. Currently, there are 14 approved indications for HBOT are represented in Table 1. Most of these uses, can be grouped according to three main effects (a) in the wound healing acceleration and angiogenesis enhancement (b) exerting antimicrobial effects, and (c) as a medical emergency.

### 4.1. HBOT and Wound Healing: The Angiogenesis Enhancement

In clinical practice, it has been observed how HBOT can speed wound healing. As wounds need oxygen to regenerate tissues properly, an exposure of 100% oxygen accelerates this process. The application in this field is quite extensive, comprising microbial-infected wounds (e.g., Clostridial myonecrosis and Fournier’s gangrene), traumatic wounds, thermal burns, skin grafts, radiation-induced wounds, diabetic and vascular insufficiency ulcers [45].

In the field of diabetes, there is a critical complication called “diabetic foot ulcers”, an open wound at the bottom of the foot that affects 15% of patients. HBOT has been specially regarded for this injury, being implicated many inflammatory and tissue repairing parameters. For instance, there was some evidence that HBOT may improve the healing rate of wounds, by increasing nitric oxide (NO) levels and the number of endothelial progenitor cells, in the non-healing vasculitis, calcific uremic arteriolopathy (CUA), livedoid vasculopathy (LV), pyoderma gangrenosum (PG) ulcers [46]. Some trials show a prominent angiogenesis while reducing inflammation: angiogenic markers like epithelial growth factor (EGF) and VEGF become enhanced, and positively associates to Nrf2 transcription factor increase [47]. Furthermore, anaerobic infections have a lower occurrence and amputation rates immensely decrease [48,49]. Different systematic reviews support the adjuvant use of systemic but not topical HBOT in the wound healing of diabetic foot ulcers [50,51]. However, studies results are quite heterogeneous, and it is still necessary to define which group of patients may benefit most from this intervention [52]. For instance, patients with diabetic foot ulcers and peripheral arterial occlusive disease may not improve wound healing [53]. Another recent study demonstrated that the use of HBOT may be associated with improved six-year survival in patients with diabetic foots [54]. Further studies and greater samples are required to identify the most suitable candidates for HBOT.

Additionally, HBOT may be an excellent adjuvant in surgery injuries resolutions, and it is key as it may provide better outcomes if it is earlier administered. When wounds do not follow conventional treatments for healing, an extra aid can be found in HBOT. Animal models have described the importance of this procedure in the wound healing by the acceleration of epithelialization and neovascularization [55,56]. Reported effects on these events resides in the up-regulation of host factors like tumour necrosis factor-α (TNF-α), matrix metallopeptidase 9 (MMP-9) and tissue inhibitor of metalloproteinase-1 (TIMP-1) [57]. In a rabbit model of irradiated tissue, NBOT O_2_ was compared to hyperbaric demonstrating once again that O_2_ is required at higher pressures to provoke an angiogenic effect [56]. More studies in vivo have alleged tension exerted by hyperbaric O_2_ modulates proliferation rate of stem cells in small intestinal crypts and raises angiogenesis in chorio-allantonic membrane in *Gallus gallus* embryos [58]. In a clinical trial of patients with chronic non-healing wounds (more than 20 months without healing), HBOT was standardized for 20 sessions (five sessions/week). The results were increased levels of vascular endothelial growth factor (VEGF) and interleukine-6 (IL-6), and lower levels of endothelin-1. These facts entail an activation of host wound resolution factors, angiogenesis and vascular tone [59]. Vasculogenesis gains efficiency thanks to HBOT upregulation of nitric oxide (NO) and associates to a decrease in lesions area [60].

Multiple lines of research have also been opened to evaluate the enhanced angiogenesis and healing of tissues following HBOT. For instance, a phase 2A clinical trial demonstrated the possible benefits from HBOT in combination with steroids for patients with ulcerative colitis in terms of achieving higher rates of clinical remission, and a reduced probability of progression to second-line therapy during the hospitalization [61]. However, there are few studies in this field, and soon an updated meta-analysis and systematic review of the available evidence will be published [62]. Similar conclusions might be extrapolated to radiation-induced hemorrhagic cystitis and proctitis [63]. Osteoradionecrosis is also a frequent and worrisome condition in oncological patients after receiving radiotherapy. Frequently, this condition affects to the jaw and consists of the development aseptic, avascular necrosis which can lead to infection, tooth loss, and even pathological fracture of the jaw. Moreover, it often results in an ulceration and necrosis of the mucosa with exposed bone. HBOT plays a critical role in the treatment of this condition, improving the tissue response to surgical wounding, and even as prophylactic approach in patients with previous head and neck irradiation undergoing dental extractions or complete exodontia [64]. The enhancing angiogenesis and wound healing make HBOT an adequate adjuvant treatment in a wide variety of conditions, although future studies should be directed to evaluate the most effective dose and to identify the most suitable candidates for submitting this procedure.

### 4.2. HBOT and Infections: The Antimicrobial Activity

The use of HBOT as an antimicrobial adjuvant is particularly useful in healing context now that microbial infections are the most important cause of non-healing wounds: meta-analysis affirm that prevalence of bacterial biofilms in chronic wounds is 78.2% [65]. HBOT is considered a non-conventional strategy for non-healing wounds consisting in a modification of biophysical parameters in the wound microenvironment, breaking the bacterial biofilms [66]. HBOT upregulates HIF that induces the expression of Nitric Oxide Synthases (NOS) and virus killing peptides (defensins and cathelicidins such as cathelin-related antimicrobial peptide) with consequent neutrophil and monocyte phagocytosis of the microbes [67,68,69]. Increased cathelicidins in mice lungs provide a better response to the flu virus [70]. Cathelicidin-deficient mice show higher susceptibility to viral damage [71].

The most important applications of the antimicrobial activity of HBOT are under necrotizing soft tissue infections (NSTIs), including necrotizing fasciitis, Fournier’s gangrene and gas gangrene. There is a calamitous soft tissue infection implying a wide variety of gram-positive, gram-negative, aerobic and anaerobic bacteria. It happens under conditions of trauma or minor lesions that become more complicated, normally, due to systemic problems like diabetes or vascular disfunctions [45,72]. An early and combined HBOT therapy plus current practices may be crucial as a lifesaving and cost-efficacy therapy, particularly in the most critical patients [73]. Clinical practice agrees on the necessity of HBOT in the event of an anaerobic infection, as anaerobic bacteria are killed by a much higher amount of pressurized O_2_ [74,75]. For instance, the use of HBOT in the anaerobic *Clostridium perfringens* bacteria is specially recommended [76]. This bacterium produces more than 20 recognized toxins. However, two toxins, alpha and theta are the main mediators of the infection caused by this agent. *Clostridium perfringens* growth is restricted at O_2_ tensions up to 70 mm Hg, and alpha-toxin production is halted at tensions of 250 mm Hg, also achieving bacteriostasis and other antimicrobial effects. Thus, recommended treatment is O_2_ at 3 ATA for 90 min three times in the first 24 h and twice a day for the next 2 to 5 days, always in combination with proper antibiotic use [77]. The anti-inflammatory potential of HBOT also aids to lessen tissue damage and infection expansion [72], also explained by a decrease in neutrophil activation, eviting rolling and accumulation of white blood cells (WBCs), hence limiting the production of ROS by neutrophils and avoiding reperfusion injury [45]. Moreover, this is observed in In vitro studies, having been demonstrated the biofilm shrinkage ability with the significant decreases in cellular load of anaerobic bacteria and fungi after HBOT [75]. A sepsis mouse model showed a significant increase in survival rate, >50%, with early HBOT compared to a control group that did not receive the treatment and was associated with lower expression of TNF-α, IL-6 and IL-10 [78]. Translation to clinical experience reports that the improvements in oxygenation follow the neovascularization, which avoid undesired events like amputation [28]. This is the case, for example, of Fournier’s gangrene, where bacteremia and sepsis are top factors of fatality, which can be avoided by adjuvant HBOT, providing much higher survival rates in clinical trials [79]. Sometimes unwanted events are underestimated until it is late and polymicrobial infection has bursted into surgical bone and joint lesions [80]. For that reason, molecular assessments of bacterial identification like mass spectrometry, are every time more accomplished to consider if HBOT is worthy for patients’ better recovering.

On the other hand, the use of HBOT might provide a central therapeutical option in the intracranial abscess (ICA). ICA presentation includes cerebral abscess, subdural empyema, and epidural empyema, and it is caused by an encapsulated infection in which the proper inflammatory response may damage the surrounding brain parenchyma [81]. The etiological agent might be bacteria, fungi, or a parasite, and it might appear as a consequence of a dissemination of previous infections like sinusitis, otitis, mastoiditis, dental infection; hematogenous seeding or cranial trauma [82]. Due to the high morbidity and mortality, along with the urgency of a non-invasive and effective method, HBOT has been proposed as a well-accepted adjunctive therapy for ICA, being regarded as a safe and tolerated method [83]. The main mechanisms by which HBOT represent an additional tool in the management of ICA resides on the impairment of the acidotic and hypoxic environment in ICAs due to the proper infection and the use of antibiotics [84]. Similarly, osteomyelitis is a chronic infection in the bone marrow frequently caused by bacteria or mycobacteria. It is a difficult condition to treat, as many antimicrobials do not penetrate in the bone properly. When this condition does not respond to the treatment or reemerge after receiving the therapy it is designed refractory osteomyelitis [85]. HBOT is a potential indication of refractory osteomyelitis as it provides synergist antibiotic activity, while enhancing angiogenesis, leukocyte oxidative killing and osteogenesis process [86]. A recent systematic review [87] reported that adjuvant HBOT provided almost a 75% of therapeutic success in patients with chronic refractory osteomyelitis, hence showing the importance of this treatment in bacterial infections. Malignant otitis externa, another infection, a necrotizing infection of the soft tissue of the external auditory canal which may rapidly cause skull base osteomyelitis may also benefit from the use of HBOT, although further studies are needed to conclude its effects [88].

Finally, some authors have also proposed a potential clinical use of HBOT as a medical emergency treatment of mucormycosis, a fungal infection [89]. Despite there still being few studies supporting its use, a compelling evidence show its potential use in a similar manner than necrotizing fasciitis, although further research is needed in this area.

### 4.3. HBOT in Medical Emergencies

Apart from the previously discussed applications, there has been further conditions in which HBOT may be considered. Some of them are designed as medical emergencies, in which the use of HBOT is an urgent indication for these patients. These are the cases of some infections above mentioned, decompression sickness, air or gas embolism, acute arterial insufficiencies such as central retinal arterial occlusion (CRAO), crush injury, compartment syndrome and acute traumatic ischemia, along with CO/Cyanide poisoning [89]. In this context, the central role of HBOT is derived from the rapid and effective response of the tissues under certain conditions that may be severe and even life-threatening [90].

A. Decompression sickness is a condition occurring due to the formation of bubbles caused by a reduction in ambient pressure that introduced dissolved gases within the body accidents. In turn, these bubbles drive to mechanical disruption of tissues, blood flow occlusion, endothelial dysfunction, platelet activation and capillary leakage. [91]. However, the term decompression sickness has been abandoned by the ECHM to be replaced by “Decompression illness” (DCI) [92], so in this article we will refer this malady as DCI. Clinical manifestations are at least one of more of the following: generalized fatigue or rash, joint pain, hypesthesia and in serious cases motor weakness, ataxia, pulmonary edema, shock and death [71]. DCI can occur in aviators, divers, astronauts, compressed air workers and, in some cases, it may appear due to iatrogenic causes [93]. HBOT/recompression therapy tables (US Navy Treatment Table 6 or helium/oxygen (Heliox Comex Cx30 or equivalent) are recommended for the initial treatment of DCI (Type 1 recommendation, Level C evidence). US Navy Treatment Table 5 can be used as the first recompression schedule for selected mild cases [94]. Therapies at higher pressure could be administered in exceptional cases, but it entails higher difficulties and risks. To maximize its efficacy, different adjunctive therapies are used in combination with HBOT including fluid administration, non-steroidal anti-inflammatory drugs and prophylactic agents to prevent venous thromboembolism events, particularly in paralyzed patients [93,95]. Overall, because of the high pressure, HBOT provide the opposite effects of the pathological mechanisms of DCIs, therefore exerting its therapeutical efficacy.

B. Air embolism. Apart from DCI, bubble formation of large arterial air embolism during operations are unusual occurrences but also ruinous and life-threatening. For bubble gas formation in veins from lung biopsy, arterial catheterization, cardiopulmonary bypass, HBOT is strictly necessary as there are no better alternatives in time. It provides tissue oxygenation by promoting gas reabsorption, and hence reduces ischemic injuries [96]. In this context, retrograde cerebral air embolism is a worrisome condition that may appear in major procedures (neurosurgery and cardiovascular operations, endoscopy), or during minor interventions (peripheral or central venous access), being particularly lethal when presented with encephalopathy [97]. The therapeutic basis of air embolism is similar to DCS, with HBOT as first-line therapy [98]. Some reports have emphasized the importance of an early HBOT, in the first 6 h since diagnosis, for this complication to obtain better outcomes, less sequelae or death rate [99]. However, there is some evidence of late benefits from its use, up to 60 h after the onset [100]. Even when there is no gas seen in on image test, patients may benefit from the use of HBOT [101]. On the other hand, recent data indicates that less cases appears to be treated by HBOT probably by the lack of belief of some physicians in HBOT, particularly in UK [102]. However, available evidence supports the use of this therapy to prevent and improve the outcome of such a dangerous condition.

C. CRAO is an ophthalmological complication caused by a permanent occlusion of the central retinal artery, mostly due to a embolus at its narrowest part that is typically associated with a sudden, massive loss of vision in the affected eye [103]. Prognosis for visual recovery is poor, as the retinal tissue is not tolerant to hypoxia, and it presents the highest oxygen consumption rate in the body at 13 mL/100 g per min [104]. As a result, HBOT is a robust indication for patients with CRAO and many studies have reported encouraging results from its use, minimum at eight sessions, with some advantages presented in comparison to other lines of treatment such as being a non-invasive method with low adverse effects [104,105,106,107]. However, and despite these benefits, HBOT is rarely offered for patients with CRAO [108], probably due to the lack of facilities in the hospital services.

D. Another approved indication for HBOT is crush injury and acute ischemia occurred as result of a trauma. Presentations of these damage vary from mild contusions to limb threatening damage, involving multiple tissues, from skin to muscles and bones. A severe consequence of trauma is the skeletal muscle-compartment syndrome (SMCS), a condition affecting both muscle and nerves [1]. Subsequently to trauma, the affected tissue will suffer from hypoxia, edema and ischemia. Here, the efficacy of angiogenesis has been also proved to be boosted by HBOT in animal models for ischemic limbs when combined with bone marrow derived mononuclear cells transplantation [109]. Some translational studies of multicenter randomized trials did not show a significant complete progress of healing [110], but in contrast, other trials showed the advantage of HBOT as adjunct for ischemic limbs when reconstructive surgery was not possible [111]. Evaluating skin peripheral circulation as well, the outcomes showed significant improvements in revascularization [112], therefore demonstrating the important role of HBOT in this condition.

E. CO poisoning is a problem that happens when household devices which use gas or coal produce CO due to an uncomplete combustion. Inhalation of this gas can be lethal and cause long-term problems particularly cognitive and brain deficits, presented up to a 40% of the patients and approximately one in three people develop cardiac dysfunction, like arrhythmia, left ventricular systolic dysfunction, and myocardial infarction [113]. To address these problems, HBOT has been applied [114] being associated to neurological sequelae reduction [115] and when applied in the first 24 h can reduce the risk of cognitive sequelae months later more efficiently [116]. In general, NBOT is immediately used after CO poisoning until HBOT is available [117]. Evidence indicates that HBOT should be considered for all cases of serious acute CO poisoning, loss of consciousness, ischemic cardiac changes, neurological deficits, significant metabolic acidosis, or COHb greater than 25% [113]. Another kind of poisoning in which HBOT has its application is cyanide toxicity. This issue appears with uncomplete combustion, this time, of materials like plastics, vinyl, acrylics, nylon, etc. HBOT is the primary treatment, but it exceeds when is combined with the antidote hydroxycobalamin, ameliorating mitochondrial oxidative phosphorylation function [118,119]. Potential uses of HBOT in a wide range of urgent conditions at least might be considered as an important tool in medical emergencies.

F. Severe anemias and idiopathic sudden sensorineural hearing loss. Despite not being considered a medical emergency, the use of HBOT is also indicated for these conditions [89]. In the first case, as Hb levels critically drops, O_2_ delivery to the tissues may be impaired. In this line, the use of 100%, hyperbaric O_2_ might solve this issue, simultaneously exerting a wide range of favorable effects in the hematological profile [120]. This could be especially important in patients who cannot be transfused for religion, immunologic reasons, or blood availability problems. Idiopathic sudden sensorineural hearing loss or acute acoustic trauma (AAT) are also important conditions in which HBOT could be a valuable tool. In fact, a recent systematic review and meta-analysis conducted by Rhee et al. [121] showed that the addition of HBOT to standard medical therapy is a valuable treatment option particularly for patients with severe to profound hearing loss and in those patients which received, at least 1200 min of HBOT. Apart from the regulation of ROS and inflammatory response, previous research has demonstrated the protective role of HBOT in the hair cell stereocilia, probably through hormetic mechanisms [122].

G. Finally HBOT can significantly improve symptoms and quality of life of patients affected by femoral head necrosis (ECHM recommendation type II level of evidence B) [123] as well as the previously mentioned NSTI, gas gangrene and urgent HBO alpha toxin neutralized

## 5. Translational and Potential Applications of HBOT

Besides approved indications, further lines of research have demonstrated the potential applications and translation of HBOT in the field of inflammatory and systemic conditions, cancer, COVID-19 and other conditions are summarized.

### 5.1. HBOT and Inflammation: Immunomodulatory Properties

HBOT might also be applicated in the regulation of inflammatory responses and its derived complications. Among the most important immunomodulatory effects, HBOT drives an alteration in CD4+:CD8+ ratio, a reduced proliferation of lymphocytes, and an activation of neutrophils with migration to hyperoxic regions [124]. Thus, HBOT might be used in a wide variety of conditions presenting an altered immune system as part of its pathogenesis. In this sense, it has been proposed the role of HBOT in the management of autoimmune diseases (ADs). A study conducted by Xu et al. [125] observed the overall effect of HBOT in general immune populations and particular Th1 and B lymphocytes subsets, proving its promising role in certain ADs. Furthermore, long-term exposure to HBOT was proven to supress the development of autoimmune symptoms, including proteinuria, facial erythema and lymphadenopathy [126]. In the same manner, the use of HBOT in early and middle stage of disease mice also show a significant increase in survival with a decrease in inflammatory cells, anti-dsDNA antibody titers, and amelioration of immune-complex deposition in comparison to later stage of disease [127] The use of HBOT has also proven its efficacy on rheumatoid arthritis, particularly due to the polarization of Th17 cells to T reg, with a significative reduction of cell hypoxia [128].

Similarly, these results could be extrapolated to other inflammatory conditions. For instance, HBOT provides an anti-inflammatory response in DSS-induced colitis. Through direct effects on HIF, HBOT induces antioxidant expression and the downregulation of proinflammatory cytokines like IL-6, therefore reducing colonic inflammation [129]. In vitro studies with lymphocytes from type 1 diabetes mellitus have proved effects of HBOT on inducible NOS expression, observing lower activity with a consequent decreased levels of NFkB [130]. Additionally, HBOT comprises another potential approach regarding musculoskeletal dysfunctions. Fibromyalgia represents an incapacitant disorder characterized by a widespread muscle and joint pain, frequently accompanied by systemic symptoms including cognitive dysfunction, mood disorders, fatigue, and insomnia [131]. HBOT exerts direct effects on brain activity, chronic pain and immune dysregulation, therefore improving quality of life of affected patients [132]. Interestingly, Woo et al. [133] also observed that HBOT could be considered an interesting alternative to attenuate exercise-induced inflammation and muscle damage.

Overall, previous research has indicated the favourable effects of HBOT in the immune system and also on the whole body.

### 5.2. Role of HBOT in the COVID-19 Pandemic

COVID-19 pandemic has challenged healthcare systems worldwide, overloading them with a huge burden in economy and our normalcy [134,135]. The urge of conducting massive vaccination programs besides finding better therapies for clinical management, have been the focus these months. In this context, HBOT has been proposed as an adjuvant for clinical practice in severe patients, and also for recovery after SARS-CoV-2 infection. Results from clinical trials have already demonstrated the potential uses of this treatment to redirect O_2_ diffusion avoided by hypoxemia, and its ability to eliminate inflammatory cytokines.

Nevertheless, not only hyperbaric O_2_ may be worthy for severe patients, but also for treating the named “silent” hypoxemia in those patients that do not have a bad clinical course yet [136]. This silent hypoxemia is not characterized by typical respiratory distress in critically ill patients, but it may be dangerous if it is not sooner detected as a prompt deterioration can occur without noticing [137]. In fact, previous studies have demonstrated the association between hypoxemia with fatal outcomes in patients with COVID-19 [138]. In the same manner, physicians observed that patients exhibit hypoxemia without dyspnea, being crucial to find care solutions to anticipate a problem with more patients at important risk [139]. Some cases of people with mild or even without symptoms, that contracted multi-organ failure and then died, have emphasized the importance of self-monitoring of pulse oximetry, which typically presents reduced readings in these patients [140]. Collected data from patients that did not present problems of breathing at admission, agreed with the suggestion of utilizing pulse oximetry to predict the outcome of hypoxemia/hypocapnia syndrome that defines asymptomatic hypoxia [141]. Steps forward in the understanding of our complex respiratory system have also launched reviews about the higher oxygenation rate in prone position, concerning variables like gravity, lung structure and the higher expression of nitric oxide (NO) in dorsal lung vessels than in ventral ones [142]. It has been demonstrated that HBOT increases the production of NO and ROS/RNS, inhibiting SARS-CoV-2 replication in previous In vitro models [41].

Moreover, all these facts have shed a light on finding better treatments to prevent fast hypoxia, fatality or even the need for mechanical ventilation [143,144] being HBOT a suggested adjuvant for its promising outcomes from previous animal models and clinical cases of sepsis and inflammatory diseases [145]. Preliminary comparisons of HBOT applications in COVID-19 to other maladies, like livedoid vasculopathy, have exposed the possible mechanisms that may occur: anti-inflammatory actions (decreased ICAM-1, proinflammatory cytokines and neutrophil rolling), anticoagulant actions (boosted fibrinolysis and increased plasminogen activator) and tissue healing actions (increased fibroblasts and stem cells) [146].

First studies in a severe patient affirmed that, compared to normobaric oxygen supply, the better empiric outcome agreed with the theoretic expectance of the potential uses of HBOT in COVID-19 [147]. Although it is still being evaluated scientifically, positive results are arising for COVID-19 treatment, finding an attenuation of the innate immune system, and increasing hypoxia tolerance [148]. In every report, this therapy has been rated as a potential support in the relieving of cytokine storm [149]. Now that mechanical ventilation may be long lasting and, preferably, avoided, in a controlled trial, safety and efficacy of HBOT for COVID-19 patients was successfully evaluated [150]. Another preliminary study showed rapid alleviation of hypoxemia from the beginning of the treatment in patients with COVID-19 pneumonia [151].

Anatomically, pathologic examinations of lung with early-phase COVID-19 have shown edema, proteinaceous exudate, inflammatory cellular infiltration, and interstitial thickening that entails a disproportional gas exchange. This is due to CO_2_ diffuses through tissues much faster than O_2_, about 20 times, what leads to hypocapnia [152]. Alveolar structure is altered in the COVID-19 patient, there is also hyaline membrane formation, there is thickness in alveolar membrane and the space for the diffusion of oxygen generates a lot of exudate and inflammation. Hence, diffusion from the alveolus through the haemato-alveolar membrane does not occur correctly, the concentration of oxygen in the blood and in the tissues begins to fall and the exchange of the dioxide also becomes difficult. Due to possible viral interactions with Hb [153] and a hypoxemia-induced shift in the oxyhemoglobin dissociation curve to the left, there is O_2_ saturation but low arterial blood pressure [154].

Clinical evidence from few studies about COVID-19 patients undergoing HBOT, notes that this therapy may make possible to contribute to reverse hypoxemia and ameliorating the pulmonary capillary circulation diffusion despite the thickness in alveolar membrane in disease. According to Henry’s Law, HBOT allows to increase pressure of O_2_ in the alveoli above ambient pressure. In this way, there will be a large increase of O_2_ diffusion into the pulmonary capillary circulation, more than 10 times, for its arrival in the plasma and reach the tissues independently of Hb. There will be a gain of O_2_ supply to the tissues mediated by the increase in pressure. Experimentally, hematological, biochemical and inflammatory parameters were significantly improved after HBOT. In first trials the observation of lymphocyte count was increased, whereas lactate and fibrinogen were decreased [147,151]. However, during this procedure patients may suffer from desaturation reflexes. Despite the etiology of this reflex is unclear, it might be probably caused by a vasoconstriction affecting the pulmonary arteries, due to the oxidative stress as well as direct damage in type II pneumocytes and thrombus associated with COVID-19 [124].

Notwithstanding the ongoing clinical trials and the efforts of standardize better protocols for safety, COVID-19 is not yet an accepted indication for HBOT, but this may be recommended for post-viral sequelae [155]. In order to guarantee its beneficial effects, there is still a need of more controlled trials to measure different inflammatory and hematological parameters that demonstrate that exudate and inflammation are reduced besides the improvements in alveolar circulation diffusion. This would confirm the potential of this adjuvant, also for considering the financial investment in hyperbaric chambers in hospitals.

### 5.3. HBOT and Cancer

Cancer is a complex entity which encompasses a broad spectrum of unique pathologies that share the following hallmarks: Immune system evasion, tumor-promoting inflammation, genome instability, enabling replicative immortality, activating invasion and metastasis sustaining proliferative signaling, evading growth suppressors, resisting cell death, inducing angiogenesis, and metabolic reprogramming [156]. Tumor-hypoxia plays a central role in many of these carcinogenic features, promoting an aggressive phenotype besides limit the effectiveness of radiotherapy, chemotherapy, and immunotherapy thereby worsening prognosis in the oncological patients [157]. Thus, targeting tumoral hypoxia and its downstream effectors have been proposed as a potential therapeutical approach in cancer management [158,159,160]. In this line, accumulating evidence supports the role of HBOT in the inhibition of tumor growth and therapy success, by three main mechanisms: (1) By limiting cancer-associated hypoxia, (2) through the generation of ROS and RNS and (3) restoring immune function [161]. Actual investigations show the promising role of HBOT in a wide variety of malignancies, including breast cancer, prostate cancer, head and neck cancer, colorectal cancer, leukemia, brain tumors, cervical cancer and bladder cancer [162]. Main applications derived from HBOT in oncology may be (a) As part of the treatment (b) as a radiotherapy adjuvant and (c) as a chemotherapy adjuvant [163].

The use of HBOT as part of the cancer therapy is not currently an approved indication, although some promising results have arisen recently. In this context, Thews & Vaupel [164] compared the efficacy of NBOT (1 atm) versus HBOT (2 atm) oxygenation reporting broader reductions of hypoxia under hyperbaric conditions. However, even at high pressure oxygenation, tumor hypoxia was not completely removed, hence showing that HBOT alone efficacy is limited. Importantly, as previously described HBOT was associated with increased angiogenesis, these effects are not significative in tumour cells, so its use could be important in the cancer management [165]. Conversely, a study conducted by Pande et al. [166] revealed that notwithstanding HBOT-treated mice initially induced a decrease in tumor progression, a tumorigenic effect was observed post-therapy, probably due to impaired DNA repair, mutagenicity and chromosomic aneuploidies together with an altered blood supply and nutrients. On the other hand, some authors suggest that the lack of therapeutical efficacy of HBOT might be due to the difficulty on creating a hyperoxic environment in the tumor and that, by combining HBOT with other methods it could act a as a potential cure in certain types of cancer. In this line, Lu et al. [167] proposed a combined use in prostate cancer patients of HBOT with ultrasound guided transrectal prostate puncture, in order to create a hyperoxic environment within the tumor, which may lead to DNA damage and a detention in the G2/M cycle, hence establishing the basis for future research. Similarly, tumor hypoxia is associated with the metabolic reprogramming of tumour cells, also known as the aerobic glycolysis or “Warburg effect”. This consists of a glycolytic switch of cancer cells, which refrain from performing oxidative phosphorylation [168]. In this sense, Poff et al. [169] described the combined effects of HBOT in combination with ketogenic diet in a murine model, preventing tumoral metastasis while expanding overall survival. Furthermore, HBOT alone or combined with low glucose and ketone supplementation also exert multiple benefits against late-stage metastatic cancers, by increasing the production of ROS and oxidative stress [170]. Despite the encouraging results, further research is required to establish the efficacy of HBOT in the different types of cancer, also searching for the most adequate use of this therapy in a global context.

Radiotherapy (RT) is a central component in cancer management, with approximately 50% of patients receiving this therapy contributing up to a 40% of curative success for cancer [171]. Through ionizing radiation, it creates a ROS and RNS overproduction, leading to double strand breaks, chromosomal aberrations and rearrangements with subsequent cell death or dysfunction, thus exerting its anti-tumoral effects. The effect of HBOT on human glioblastoma (GBM) was investigated, in laboratory, on patient-derived cells and on microglia cell biology (CHME-5). The results obtained from the combination of HBO and RT clearly showed a radiosensitising effect of HBO on GBM cells grown [172]. Hypofractionated stereotactic radiotherapy (HSRT) after HBO (HBO-RT) appears to be effective for the treatment of recurrent high-grade glioma (rHGG), as pointed out on a cohort of 9 adult rHGG patients. It could represent an alternative, with low toxicity, to systemic therapies for patients who cannot or refuse to undergo such treatments [173]. However, although non-tumour cells are less sensitive, radiation could also affect them, altering multiple cellular signaling pathways or inducing apoptosis, hence explaining its multiple adverse effects [174] One of the most severe consequences resulted from irradiation is the appearance of post-radiation injuries, a process starting during radiotherapy that involves the dysregulation of multiple bioactive compounds, particularly fibrogenic cytokines like TGF-β [175]. Similarly, almost all tissues with delayed irradiation injury present a histological feature named as obliterative endarteritis, finally leading to a tissue damage characterized by hypoxia, hypovascularity and hypocellularity [176]. In this line, HBOT has consistently demonstrated its therapeutical effectivity against radiation-induced injury also approved by the UHMS [177] and the ECHM [8]. Last 2016 Cochrane review [178] evidenced that the use of HBOT in head, neck, anus and rectum injured tissues were associated with improved outcomes and, at some extent with osteoradionecrosis following tooth extraction in an irradiated field. According to ECHM recommendation the use of HBOT is recommended in the treatment of radiation proctitis (Type 1 recommendation, Level A evidence), mandibular osteoradionecrosis and haemorrhagic radiation cystitis (Type 1 recommendation, Level B evidence) and suggested in the treatment of osteoradionecrosis of other bone than the mandible, for preventing loss of osseointegrated implants in irradiated bone and in the treatment of soft-tissue radionecrosis (other than cystitis and proctitis), in particular in the head and neck area (Type 2 recommendation, Level C evidence). Furthermore, it would be reasonable to use HBOT for treating or preventing radio-induced lesions of the larynx, in the treatment of radio-induced lesions of the central nervous system (Type 3 recommendation, Level C evidence) [8]

Finally, the combined use of HBOT plus chemotherapy have reported certain benefits. In this line, a recent study conducted by Brewer et al. [179] demonstrated the effectiveness of using HBOT to prevent chemotherapy-induced neuropathy In vivo. This fact appears to be due to the various implications of HBOT in the neuronal activity and signaling [180,181,182] Kawasoe et al. also observed [183] that an integrative strategy of carboplatin plus HBOT significantly reduced mortality in C3H mice with inoculated osteosarcoma cells Similar results were obtained with HBOT and chemotherapy in lung cancer cultures and animal models [184]. In particular, the combination of paclitaxel and carboplatin plus HBOT and hyperthermia show promising results for treating patients with non-small cell lung cancer and multiple metastasis [185]. Despite these results, the use of HBOT and chemotherapy may also represent a contraindication for the patients. For instance, the combination of HBOT with doxorubicin, bleomycin, or cisplatin may exert synergic cardiotoxicity, pulmonary toxicity or impaired wound healing, respectively [186]. This is an important issue to address in the oncologic patient. In these cases, it is important to separate chemotherapy from the use of necessary HBOT, to avoid undesired effects. In addition, further strategies could be considered targeting tumour hypoxia and functioning as therapeutic adjuvants like physical activity [187]. Overall, the benefits of HBOT in cancer management is a potential field to keep on exploring.

### 5.4. Other Applications

In the same manner, other novel lines of research are exploring potential uses of HBOT in a plethora of conditions. For instance, some studies related to microvascular or macrovascular insufficiencies causing erectile dysfunction (ED) have hypothesized the effects of HBOT in patients with this problem. Empirical data suggests that it can induce penile angiogenesis and improve erectile function in men suffering from ED. This is due to vasodilatation relies on proper blood vessels in corpora cavernosa. Then, being a major concentration of oxygen in tissues, there is an increased angiogenesis by VEGF and endothelial cells differentiation [188]. This application has not provided significant data on rehabilitation after prostatectomy [189] but it has obtained good symptoms resolution for other clinical manifestations like ED in diabetes mellitus [190] or in recovery after urethral reconstruction [191]

Equally, the use of HBOT for ischemic stroke and brain injury is an interesting point of study. For instance, different studies have demonstrated the importance of this procedure as a prophylactic approach for sequestration of inflammation inherent in stroke and traumatic brain injury, preventing neuronal death [192]. Other uses such as brain preconditioning before stem cells transplantation have also been explored [193]. However, the efficacy and safety of HBOT in these conditions remains to be fully elucidated, although some basic and clinical research have shown encouraging results [194].

Finally, the use of HBOT could be potentially extended to novel fields like aging. Hachmo et al. [195] reported the effect of hyperbaric oxygen in the prevention of telomere shortening and immunosenescence by the clearance of senescent immune cells. In this line, other studies have reported the same results in the aging skin, through the acceleration of epidermal basal cells proliferation [196], in the endothelial cells, where it induces antioxidants expression [197] and also in the brain, where HBOT appears to improve the cerebral blood flow [198], restoring cognitive parameters, hippocampal functions and even improved insulin resistance in both normal-weigh and obese aging rats [199].

**Figure 2:**
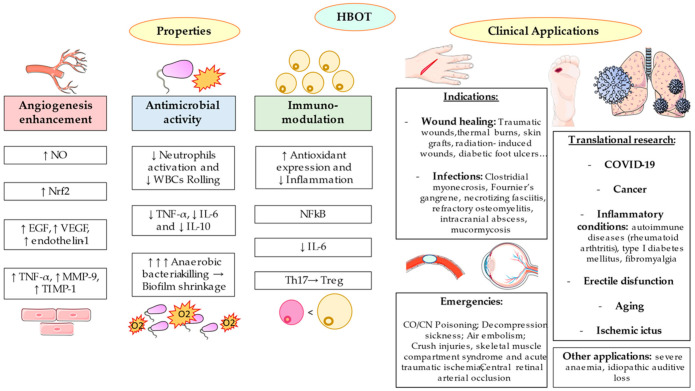
Summary of top properties of HBOT and its clinical applications. Firstly, it can provide an angiogenesis enhancement, observed by the prime production of NO which subsequently brings an upregulation of Nrf2 and growth factors like epidermal growth factor (EGF), vascular endothelial growth factor (VEGF) and endothelin-1. TNF-α, matrix metallopeptidase 9 (MMP-9) and tissue inhibitor of metalloproteinase-1 (TIMP-1) will be boosted too. Secondly, the antimicrobial activity is visible due to bacterial killing by O_2_, removing biofilm and lessening white blood cells (WBCs) rolling and neutrophils recruitment, hence promoting a downregulation of proinflammatory cytokines (TNF-α, IL-6 and IL-10). The immunomodulation properties are observed by a downregulation of transcriptional factor NFkB, involving a proinflammatory response switch off (IL-6) and a polarization from Th17 lymphocytes to Treg. Summarized applications include: indications for which HBOT is approved (mostly wound healing and infections), primary emergencies (like CO/CN poisoning or air embolism), and translational research (comprising COVID-19, cancer, inflammatory conditions or aging among others).

As summarized in Figure 2, the main consequences of HBOT and its related hyperoxemia and hyperoxia in the human body could be related with the angiogenesis enhancement, antimicrobial properties and immunomodulatory effects. Approved indications for this therapy could also be grouped according to its emergency.

## 6. Adverse Effects and Contraindications

Notwithstanding the multiple benefits and applications of HBOT, there are important adverse effects that may appear during this procedure. As a result of the hyperoxia and the hyperbaric environment, there are some issues when using this therapy. The two most common complications during HBOT are claustrophobia and barotrauma. Both occur during monoplace or multiplace chamber compression [200]. In the case of barotrauma, it could be defined as an injury caused by an inability to equalize pressure from an air-containing space and the surrounding environment. Ear barotrauma is the most frequent condition affecting the middle ear, although sinus/paranasal, dental or pulmonary barotrauma could also be reported [201]. Despite the incidence of this complication being extremely rare [202], its seriousness should be taken into account, considering clinical history of patients at risk of suffering from these complications while implementing different strategies to prevent this complication, such as anti-epileptic therapy, prolonged air brakes or controlling treatment pressure [203]. The last event is associated with the appearance of the Paul Bert effect because of the formation of seizures that may bring transient but negative consequences for cognitive functioning and behavioural patterns [204]. These effects are primarily due to the toxic properties of oxygen at high concentrations. However, to date, no threshold has been described to precisely assess the pathological levels of oxygen, which could be an important issue for critical patients [205]. Pulmonary toxicity is not associated with the use of repeated hyperbaric oxygen following current protocols [206]. Ocular manifestations from HBOT may also be described, particularly hyperbaric myopia, transitory in most cases. Other ophthalmological complications less frequent observed are cataracts, keratoconus or retinopathy of prematurity, in the case of pregnant women exposed to HBOT [207,208]. All these adverse effects may be ameliorated prominently by an adequate screening, through the use of certain devices and the adjustment of the treatment protocols [200,201]

On the other hand, there are certain conditions in which HBOT might be absolutely contraindicated or relatively contraindicated. The first case is exclusively represented by untreated pneumothorax, as it could be a life-threatening procedure [209]. The rest of contraindications are relative, its indication will depend on the real necessity of this therapy. Aside from the chemotherapheutic agents previously described other treatments like sulfamylon (Mafenide), could also share the same action than cisplatin impeding wound healing effects derived from HBOT, and it should also be interrupted before this therapy [45]. If patient has a pacemaker or any type of implantable devices, it is necessary to verify its safety with increased pressure or with pure concentrations of oxygen. Hereditary spherocytosis may also be a contraindication, as hyperbaric oxygen could cause severe haemolysis [43]. Pregnancy is another potential contraindication for this therapy in exception of CO poisoning [210]. Although rare in non-diabetic individuals, patients may also suffer from hypoglycaemia during this procedure, and it is important to evaluate their blood glucose levels before HBOT, as it could aggravate their hypoglycaemic profile [211]. Similarly, patients with underlying respiratory pathologies like chronic obstructive pulmonary disease (COPD), asthma and even upper respiratory infections might be also possible contraindications from receiving HBOT, as it could increase the risk of hypercapnia, pulmonary barotrauma and sinus or middle ear barotrauma, respectively [209]. An additional effect derived from HBOT is the increment of blood pressure [212]. Hyperbaric oxygen may also induce pulmonary oedema and cardiovascular difficulties in patients with heart failure or in patients with reduced cardiac ejection fractions [213]. Finally, the history of epilepsy, hypoglycaemia, hyperthyroidism, current fever, and certain drugs such as penicillin and disulfiram are also thought to lower the seizure threshold during this therapy [214]. Diabetic patients may be warned from regulating its doses of HBOT in order to prevent the hypoglycaemic effect of this therapy.

To summarize, despite the multiple applications of HBOT it is equally important to consider its potential adverse effects and underlying conditions in which this therapy is not going to exert its efficacy, also representing a potential risk for these patients.

## 7. Conclusions and Future Directions

HBOT is an effective method to increase blood and tissue oxygen levels, independently from Hb transportation. Its therapeutical basis could be understood from three different perspectives: Physical (Hyperbaric 100% oxygen), physiological (Hyperoxia and hyperoxemia) and cellular/molecular effects. All these effects provide HBOT its efficacy in the management of hypoxia derived conditions and hypoxemia, respectively, also exerting direct effects in infectious agents and immune cells, modulating a wide variety of cellular signaling pathways, cytokine production and tissue processes such as angiogenesis. Herein, the use of HBOT might be extended to a broad spectrum of pathologies, from infections and inflammatory/systemic maladies to wound healing and vascular complications, also reporting its efficacy in the management of medical emergencies like air embolism or gas poisoning. Although respiratory infections and diseases have been mentioned as contraindications for HBOT, the case of SARS-CoV-2 is an exception. Nowadays, the potential use of HBOT in the COVID-19 has been specially regarded, exposing results in numerous controlled clinical trials. Moreover, the use of this procedure in different types of malignancies represents an important support in the delayed radiation injury. In the same manner, the use of HBOT as a therapeutical agent have shown promising results in trials as an adjunctive substance with other approved treatments like chemotherapy and even, recent research have also reported significative improvements in nanomedicine approaches when combined with HBOT [215].

Despite its benefits, there are still certain challenges which need to be overcome to improve the current and potential applications of HBOT. In this line, a worrisome issue would be to develop sophisticated strategies to address tissue hypoxia, as for certain conditions like tumoral cells, the HBOT induced hyperoxia does not completely eliminate tumour hypoxia. An adequate combination of HBOT with another procedure might be interesting to targeting this problem [167]. On the other hand, it is equally important to determine and quantify potential adverse effects derived from HBOT, as well as potential contraindications from receiving this therapy. Future research should be destinated on developing accurate systems to determine potential benefits and risks for patients before submitting HBOT. In this line, the development of predictive models as previously mentioned or novel strategies could be interesting approaches in these fields.

Currently, there are only 14 approved indications for this therapeutical approach. We encourage further studies to extend the possible uses of this procedure, always considering individual benefits and risks from receiving this therapy. The inclusion of HBOT in future clinical research could be an additional support in the clinical management of multiple pathologies.

## Figures and Tables

**Table 1:** Approved indications for HBOT.

Air or gas embolism
Acute thermal burn injury
Carbon monoxide poisoning
Carbon monoxide poisoning complicated by cyanide poisoning
Central retinal artery occlusion
Clostridial myositis and myonecrosis (gas gangrene)
Compromised grafts and Flaps
Crush injury, Compartment Syndrome and other acute traumatic ischemia
Decompression sickness
Delayed radiation injury (soft tissue and bony necrosis)
Enhancement of healing in selected problem wounds
Idiopathic sudden sensorineural hearing loss
Intracranial abscess
Necrotizing soft tissue infections
Refractory osteomyelitis
Severe anaemia

## Data Availability

The data used to support the findings of the present study are available from the corresponding author upon request.
